# Airway obstruction by a retropharyngeal hematoma secondary to thoracic aortic aneurysm rupture

**DOI:** 10.1186/1749-8090-8-232

**Published:** 2013-12-27

**Authors:** Hiroshi Kubota, Hidehito Endo, Mio Noma, Hiroshi Tsuchiya, Akihiro Yoshimoto, Yusuke Inaba, Yoshifumi Nishino, Ayaka Tsuboi, Yuki Sato, Naoyuki Kohno

**Affiliations:** 1 Department of Cardiovascular Surgery, Kyorin University, 6-20-2, Shinkawa, Mitaka, Tokyo 181-8611, Japan; 2 Department of Otorhinolaryngology, Kyorin University, 6-20-2, Shinkawa, Mitaka, Tokyo 181-8611, Japan

**Keywords:** Retropharyngeal hematoma, Airway obstruction, Thoracic aorta, Aortic aneurysm, Rupture, Shock, Aortic arch, Open-stent graft, Mediastinal hematoma

## Abstract

**Background:**

Retropharyngeal hematoma is a rare form of pharyngeal pathology and can present as acute airway obstruction. Among the many causes of retropharyngeal hematoma, thoracic aortic rupture is extremely rare.

**Methods and results:**

A 78-year-old female with airway obstruction by a retropharyngeal hematoma secondary to thoracic aortic aneurysm rupture was successfully treated by total aortic arch replacement and open stent-graft insertion.

**Conclusion:**

Rupture of the thoracic aorta should be considered as a rare but important cause of retropharyngeal hematoma and airway obstruction.

## Background

We recently encountered a patient with a distal aortic arch aneurysm and mediastinal hematoma that extended into the retropharyngeal space. The patient developed acute airway obstruction and lost consciousness. Total aortic arch replacement with open stent-graft insertion was performed. The postoperative course was uneventful. As of 16 months after the operation, the patient is alive and well and has had no recurrences.

## Case presentation

**Figure 1:**
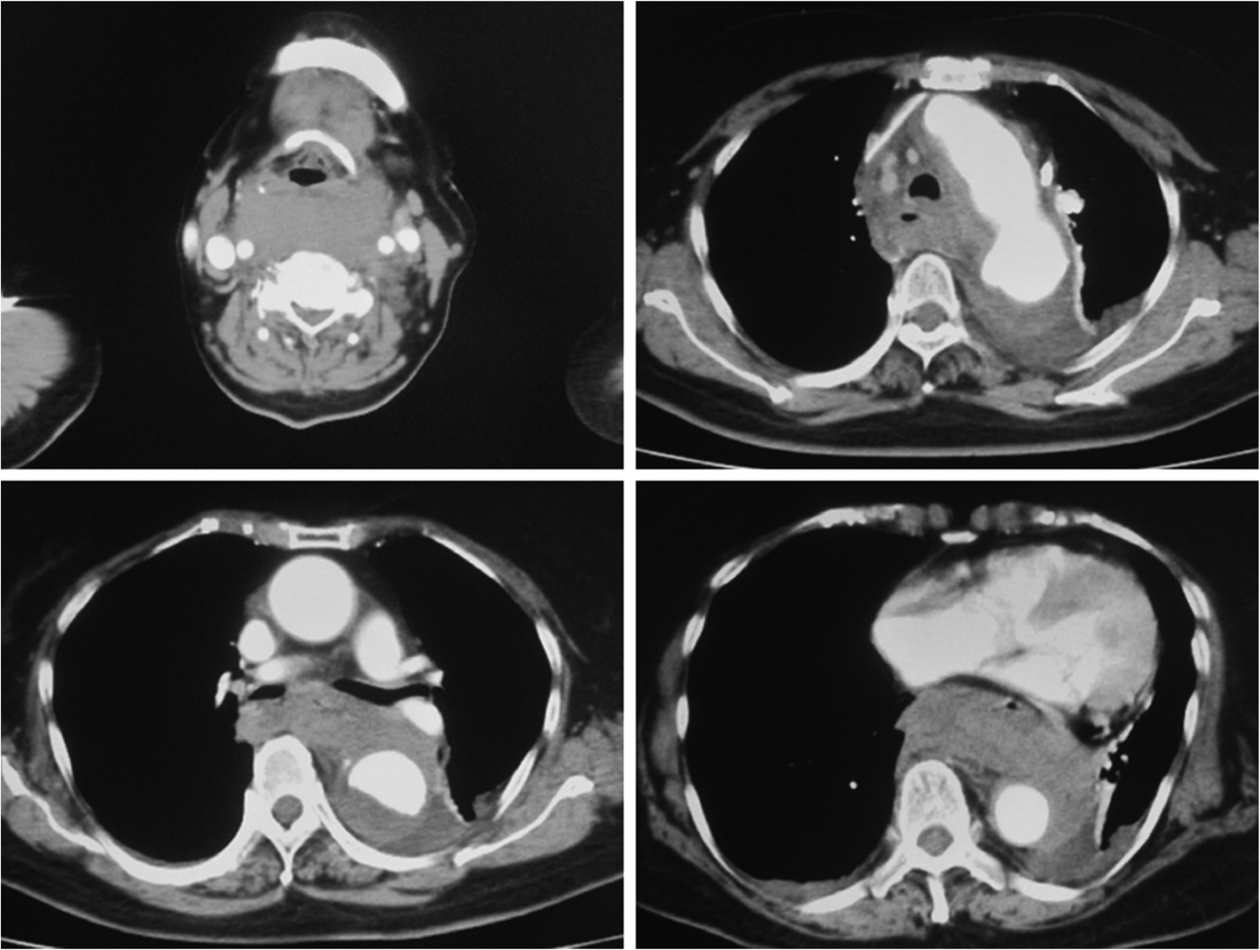
**Contrast-enhanced computed tomography scans.** The larynx was compressed and narrowed by the hematoma. A large cervico-mediastinal hematoma was detected.

**Figure 2:**
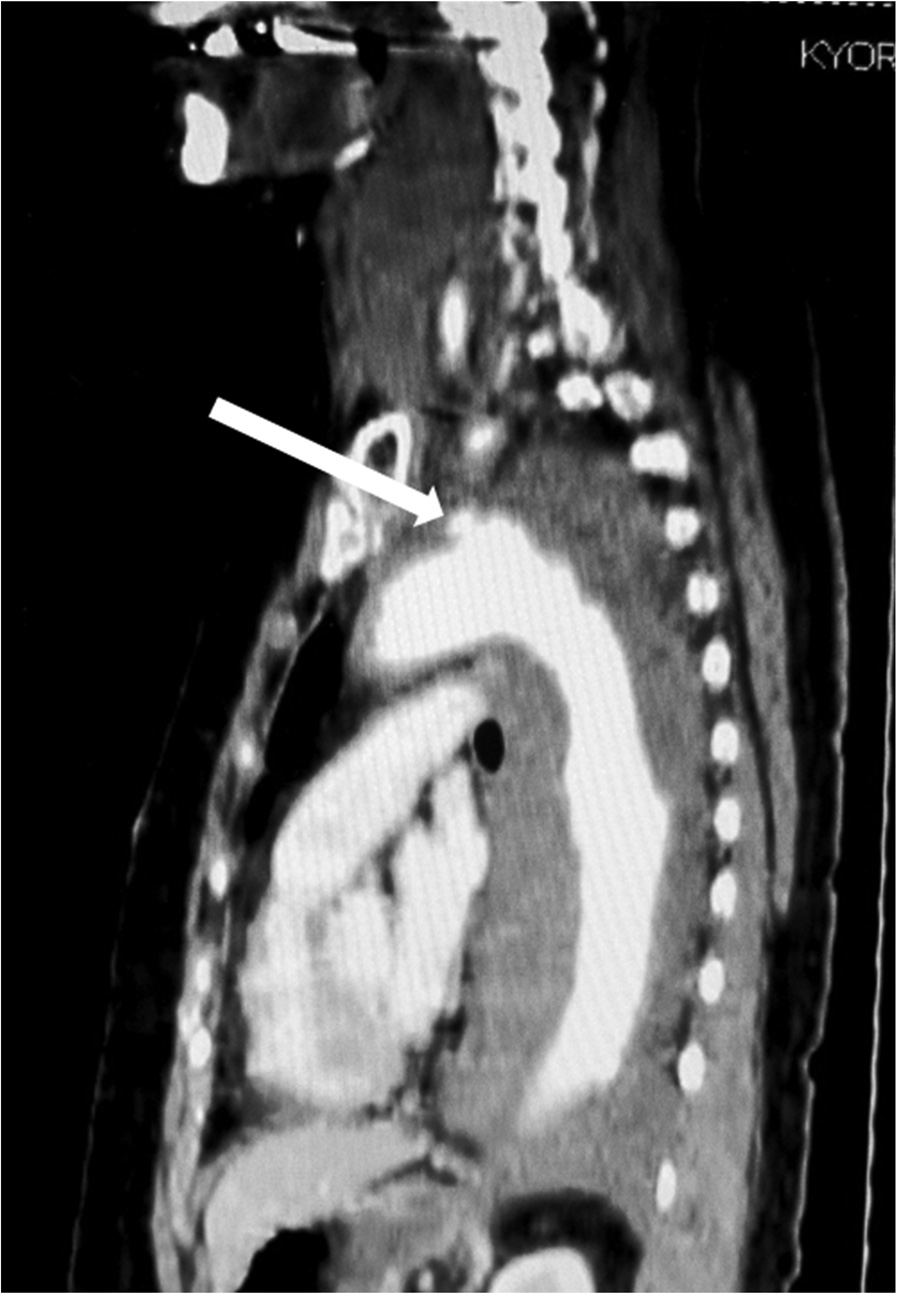
**Reconstructed contrast-enhanced computed tomography scan.** Dense atheromatous change was seen in the thoaracic aorta. A penetrating atherosclerotic ulcer was seen in the distal aortic arch (arrow).

**Figure 3:**
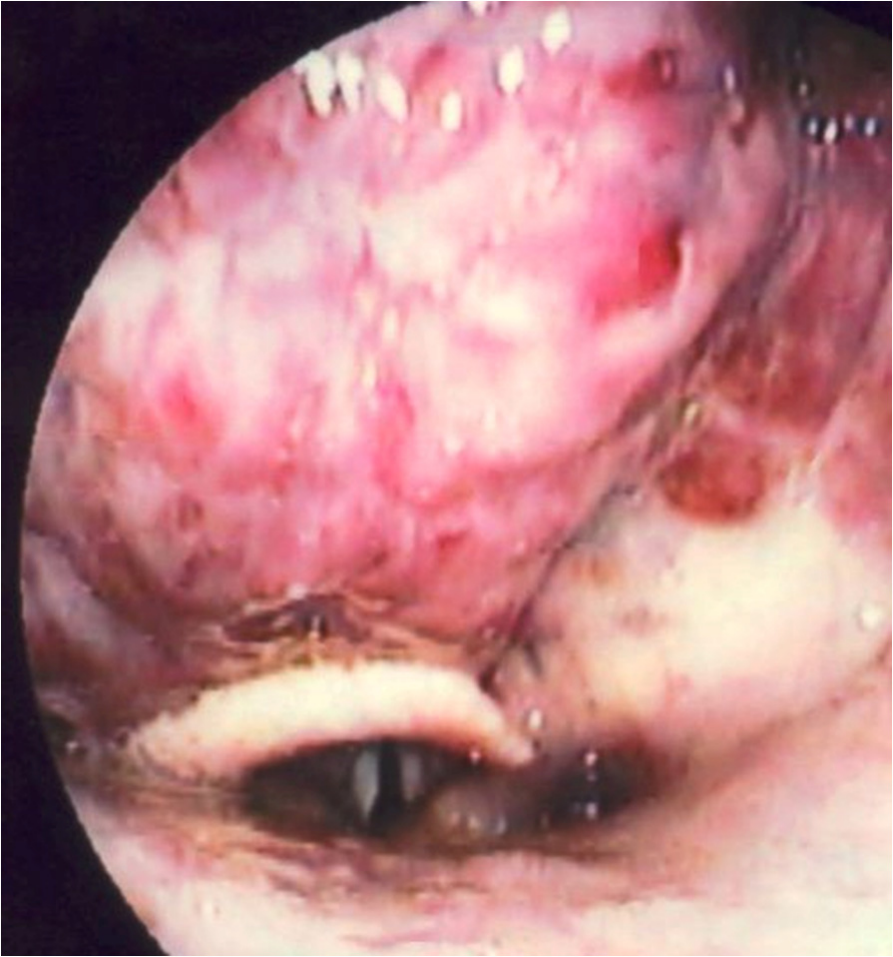
**Laryngoscopic findings.** The pharyngeal space was narrowed, and the larynx was severely compressed by the hematoma.

A 78-year-old Asian female was brought to our hospital by ambulance in July 2012. During the night she had experienced sudden dyspnea with back pain, become cyanotic, and lost consciousness. When the ambulance arrived at her home, she was still unconscious, and oxygen saturation was too low to be measured. The patient gradually regained consciousness in the ambulance en route to our hospital. Echocardiography showed left ventricular hypertrophy and normal left ventricular function. A chest X-ray showed an enlarged mediastinal shadow. Contrast-enhanced computed tomography revealed the presence of a large cervico-mediastinal hematoma and that the pharynx and larynx were severely compressed and narrowed by the hematoma (Figure 1). Reconstructed contrast-enhanced computed tomography images showed a penetrating atherosclerotic ulcer in the distal aortic arch and a dense mural thrombus in the thoracic descending aorta (Figure 2). Laryngoscopy revealed narrowing of the pharyngeal space and severe compression of the larynx by a bulging hematoma (Figure 3). The patient had a past history of hypertension, hyperlipidemia, and bronchial asthma. She was being treated for arteriosclerosis obliterans with ethyl icosapentate and beraprost sodium.

A diagnosis of airway obstruction by a retropharyngeal hematoma secondary to rupture of an aneurysm of the thoracic aorta was made, and emergency total arch replacement and open stent-graft insertion was planned. A median sternotomy was performed, and a cardiopulmonary bypass was established via both venae cavae and the proximal ascending aorta. When the patient’s tympanic membrane temperature reached 18°C, under circulatory arrest with intermittent pressure-augmented retrograde cerebral perfusion, the aorta was transected just distal to the left subclavian artery [1,2]. A dense, soft atheroma was observed inside the distal aortic arch, and it extended into the descending aorta. A large dimple was noted in the greater curvature of the distal arch, 20 mm distal to the left subclavian artery. Further exploration deep inside the dimple to detect the perforation site was avoided in order not to trigger a distal embolism. A 30-mm diameter prosthetic graft with a Z-stent (GZV 40–50; William Cook Europe, Bjaeverskov, Denmark) was inserted at the level of the 7^th^ thoracic vertebra with a CLATE stent-graft delivery system (Senko Medical Instrument Mfg., Co., Ltd., Tokyo, Japan) [3]. After placing the open-stent graft, a distal anastomosis, and the left subclavian artery and the left common carotid artery anastomoses were performed to a 4-branched 30-mm prosthetic graft. While re-warming the patient by resumed cardiopulmonary bypass by using a side branch of the graft, the brachiocephalic artery and the proximal aorta were anastomosed to the graft, and the aorta was declamped.

Before closing the chest wall, the left parietal pleura was opened, and 200 ml of a bloody effusion was aspirated. Circulatory arrest time was 68 minutes.

### Results

**Figure 4:**
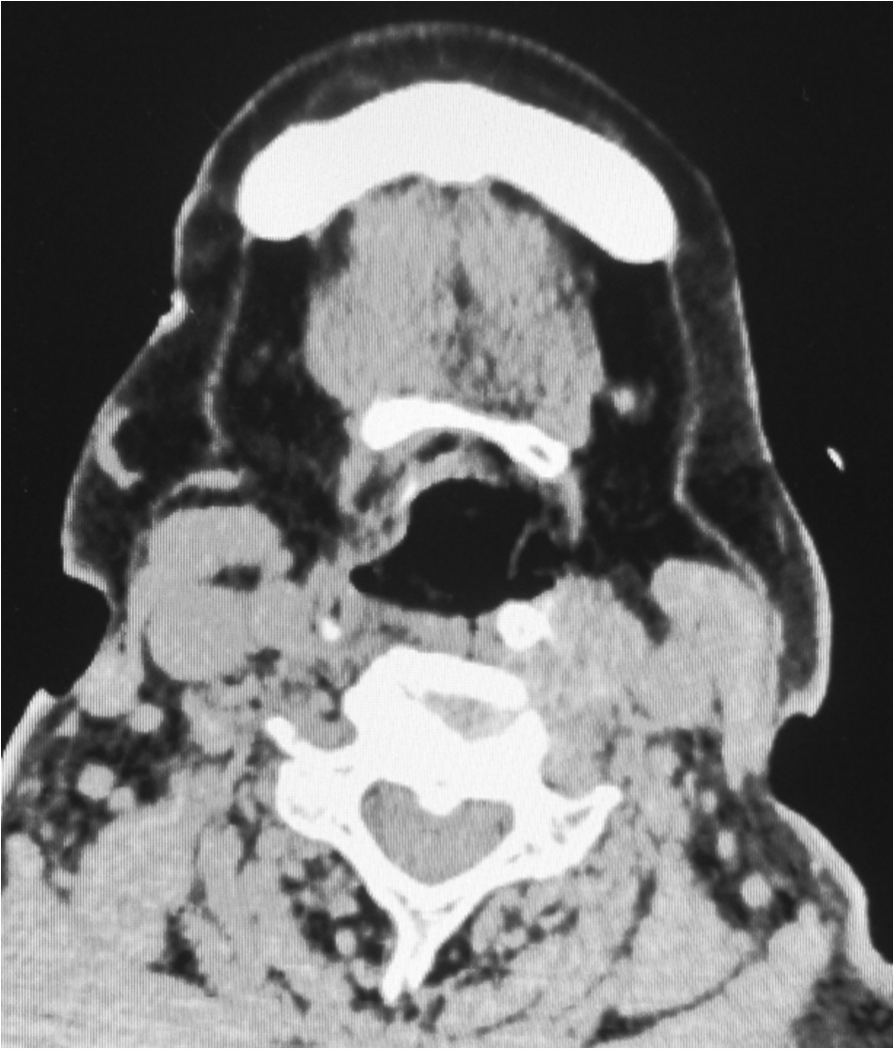
**Postoperative computed tomography scans.** The retropharyngeal hematoma was no longer seen.

**Figure 5:**
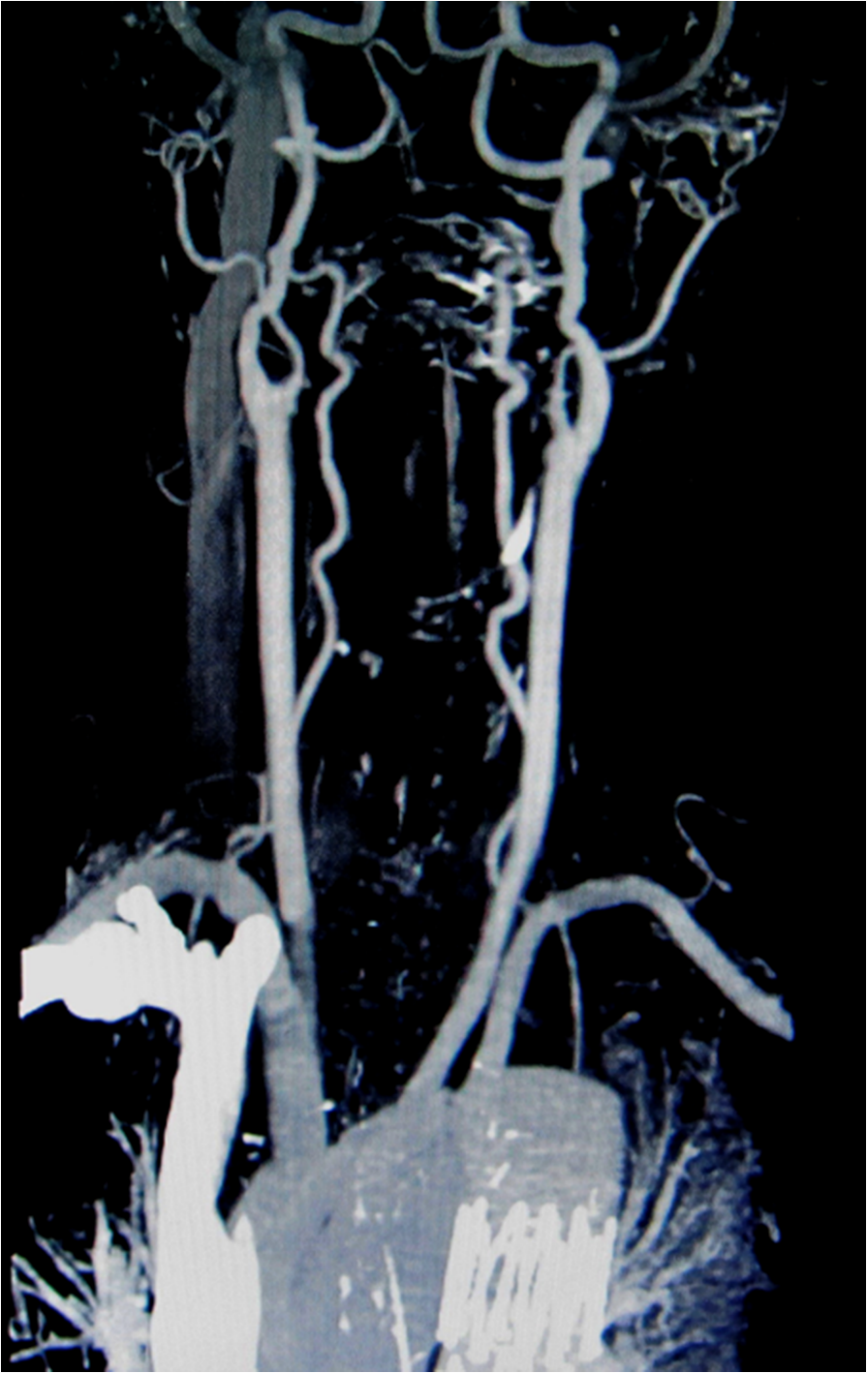
**Postoperative 3-D reconstructed computed tomography scan.** No abnormal vessels or aneurysms were detected in the neck.

The postoperative course was uneventful. After confirming regression of the pharyngeal swelling with a fiberscope, the patient was extubated on postoperative day 3. Postoperative computed tomography confirmed the absence of the retropharyngeal hematoma and regression of the mediastinal hematoma (Figure 4). 3-D computed tomography showed a well-fitting open stent graft. No abnormal neck vessels or neck vessel aneurysms were detected (Figure 5). The intercostal arteries, which were covered by the stent graft, were occluded. The patient’s back pain resolved soon after operation, and the patient is alive and well as of 16 months after the operation.

### Discussion

Retropharyngeal hematoma is a rare form of pharyngeal pathology. Retropharyngeal hematomas are caused by trauma, vertebra artery aneurysms, thyroid hemorrhages, parathyroid hemorrhages, parathyroid cysts, parathyroid adenomas, and tumors. Anticoagulation- therapy-related spontaneous bleeding is also known to cause retropharyngeal hematomas [4-9]. Mediastinal hematomas are usually caused by thoracic aortic aneurysms, aortic dissection, esophageal injury, traumatic aortic injury, bronchial artery aneurysms, intercostal artery aneurysms, and central venous catheter insertion.

Sabra et al. reported the case of a patient who presented with neck swelling and dyspnea due to rupture of the descending aorta in which the patient was rescued by a stent-graft replacement. The clinical features and computed tomography findings in their case were very similar to the clinical features and computed tomography findings in our case [10]. According to their report, only one case with the same symptoms caused by a cervico-mediastinal hematoma due to aortic rupture had ever been reported [11,12]. Thus, because of the extremely rare clinical features of our case, it was difficult to determine the cause. Based on the patient’s past history, diagnostic imaging findings, and persistent back pain, we concluded that the retropharyngeal hematoma was not the primary lesion but secondary to rupture of the thoracic aorta. We suspected that treatment with the antiplatelet drug may have accelerated extension of the hematoma. We did not attempt to identify the exact site of the rupture intraoperatively, because we wanted to perform the operation as safely and less-invasively as possible. However, the intraoperative findings and uneventful postoperative course strongly supported the diagnosis. Because there have been few reports describing retropharyngeal hematomas secondary to aortic events, the route by which aortic ruptures extend into the cervical space is unclear. However, there have been several descriptions of the anatomical features of descending necrotizing mediastinitis.

Although the extension is in the opposite direction, those descriptions provided us with some clues as to possible routes of extension from the aorta to the neck. Moncada et al. have stated that there are three primary routes of spread of infection from the neck to the mediastinum: a route via the pretracheal space, a route via the retrovisceral space, and a route via the perivascular compartment [13]. These spaces account for approximately 8%, 71%, and 21%, respectively, of the routes of spread in cases of spread from above to the mediastinum. The hematoma in our patient appeared to have ascended via the perivascular compartment. The perivascular compartment includes the carotid sheath and its neural and vascular structures. It is noteworthy that Moncada et al. stated that “involvement of this space may result in major vessel rupture and cranial nerve deficits”. We inserted an open stent-graft in addition for three reasons. The first reason was to treat the extensively dilated thoracic descending aorta. The second reason was to prevent a distal embolism by fixing the dense mural soft thrombus. The third reason was to block the intercostal arteries and bronchial arteries, which have the potential to cause a mediastinal hematoma. Because of the lower mortality and morbidity associated with thoracic endovascular aortic repair (TEVAR), it is considered an alternative to open surgical repair with cardiopulmonary bypass and systemic hypothermia. Several TEVAR procedures: fenestrated TEVAR, TEVAR with chimney grafts, and debranching followed by TEVAR are regarded as alternative approaches to the treatment of ruptures of the distal aortic arch. Because, in contrast to the dense atheromatous change in our patient’s aortic arch, there was no atheromatous change in the ascending aorta, it appears that debranching followed by TEVAR would have been a less invasive alternative procedure than other TEVAR procedures or open surgery in our patient. However, in view of the fact that the risk of thromboembolism would still have existed even if we had used the TEVAR procedure, and since we had no experience performing the debranching followed by TEVAR procedure at the time, we selected one-stage open repair with an open stent-graft as described above.

The pharyngeal swelling rapidly regressed postoperatively. The surrounding tissue edema due to impairment of the venous drainage as well as the hematoma was thought to have promoted the swelling.

The maximum diameter of the thoracic aorta in our patient was 50 mm. Davies et al. reported that the risk of rupture of aneurysms of the thoracic aorta more closely related to the “aortic size index” (diameter of the aneurysm/body surface area) than to the absolute diameter of the aneurysm. They divided patients into 3 groups: a low-risk group (aortic size index <2.75 cm/m^2^, incidence of rupture 4% a year), a moderate-risk group (aortic size index 2.75-4.24 cm/m^2^, incidence of rupture 8% a year), and a high-risk group (aortic size index >4.24 cm/m^2^, incidence of rupture 20% a year [14]. They stated that the reason female sex is a significant predictor of aortic rupture or dissection is in part attributable to the gender differences in mean body size and aortic dimensions having a proportionally greater diameter in smaller woman. The aortic size index of our patient was 3.2 cm/m^2^, and she belonged to the moderate-risk group.

## Conclusions

This rare entity, rupture of the thoracic aorta, should be considered an important differential diagnosis among the potential causes of retropharyngeal hematomas and airway obstruction.

## Consent

Written informed consent was obtained from the patient for publication of this Case report and any accompanying images. A copy of the written consent is available for review by the Editor-in-Chief of this journal.

## Abbreviations

TEVAR: Thoracic endovascular aortic repair.

## Competing interests

The authors declare that they have no competing interests.

## Authors’ contributions

HK is the primary author of the manuscript. HE, MN, HT, AY, YI, YN, AT, YS have been involved in drafting the manuscript or revising it critically for important intellectual content, and NK have given final approval of the version to be published. All authors read and approved the final manuscript.
